# Complementary mechanisms stabilize national food production

**DOI:** 10.1038/s41598-021-84272-z

**Published:** 2021-03-01

**Authors:** Lucie Mahaut, Cyrille Violle, Delphine Renard

**Affiliations:** UMR 5175 Centre d’Ecologie Fonctionnelle et Evolutive, Univ Montpellier, CNRS, EPHE, IRD, Univ Paul Valéry Montpellier 3, Montpellier, France

**Keywords:** Ecology, Agroecology, Biodiversity, Ecosystem services

## Abstract

Ensuring the temporal stability of national food production is crucial for avoiding sharp drops in domestic food availability. The average stability of individual crop yields and asynchrony among crop yield fluctuations are two candidate mechanisms to stabilize national food production. However, the quantification of their respective influence on the stability of national food production is lacking, as is the identification of the factors regulating both mechanisms. Using yield data for 138 crops and 115 countries over a 50-year period, we first show that the stability of total national yield mostly relies on the fluctuations of the yield of crops covering the largest share of cropland. The average yield stability of these crops exert a stabilizing effect on national food production that is twice as important as the one of the asynchronous yield fluctuations among them. Climate variability reduces the stability of national food production by synchronizing yield fluctuations among crops and destabilizing the yield of individual crops. However, our results suggest that increasing crop diversity can counteract the synchronizing effects of climate variability by enhancing asynchronous dynamics among crops. Irrigation can promote the average stability of individual crop yields but cannot compensate for the destabilizing effect of climate variability. Considering both the response of each crop to climatic variations and the dynamics emerging from crop baskets will help agricultural policies to ensure stable food supply at the national level.

## Introduction

Maintaining the year-to-year stability of national food supply is a critical target for agricultural policies to support food security worldwide. The increasing frequency of extreme climate events such as drought led to strong fluctuations in food production at the national (e.g.^1^) and global^2–4^ scales. Such fluctuations in crop production can adversely affect the stability of food supply and imped food security^5^. Increasing domestic grain reserves^6^ and importing food through international trade^7–9^ can compensate for sharp drops in domestic food availability. However, grain reserves are declining worldwide^8^ and international trade could be source of instability and threaten food sovereignty and security in low-income nations^10^. Moreover, with increasing synchronization of major crop failures at the global scale^4^, relying on food imports to ensure food supply seems a limited solution in the long term. In this context, increasing the stability of national food production is a promising, alternative solution to safeguard society against food shortages. Achieving this goal requires the investigation of the different levers of action and of the mechanisms leading to greater stability of national food production.

Decades of research in ecology show that the year-to-year stability of ecosystem productivity results from two distinct mechanisms: the stability of each individual species and the asynchronous variation among species that allows for losses and gains in productivity to compensate each other (i.e. asynchronous dynamics^11^). In cropping systems, the use of agricultural inputs such as irrigation water and fertilizers is one way to buffer the effect of environmental fluctuations and stabilize individual crop yields (e.g.^3,12^). However, ecological experiments in grasslands show that nutrient enrichment reduces the stability of ecosystem productivity by synchronizing the fluctuations among plant species (e.g.^13–15^). Investigating whether and how agricultural inputs affect asynchronous dynamics among crops is therefore a critical issue to understand the overall effects of agricultural inputs on the stability of national food production.

Another promising strategy to stabilize total national yield is to increase the diversity of crop species or crop groups grown within a country^16^. However, the mechanism underlying the crop diversity-national yield stability relationship remains to be elucidated. Theories and experiments in biodiversity-ecosystem functioning research (BEF) show that increasing plant diversity enhances asynchronous dynamics (reviewed in^17,18^). While the role of species diversity in asynchrony has been extensively studied at the local scale, where species interact (e.g.^19^), recent theoretical developments predict that these dynamics can also play out across larger spatial scales^20,21^. Importantly, attaining greater yield stability through higher crop diversity will also require identifying crops with asynchronous dynamics. Renard and Tilman^16^ provide evidences that the stabilizing effect of crop diversity on national food production depends on the number of crops representing an equal share of harvested area (‘effective’ diversity) and not simply on the total number of crops grown in a country. This suggests that fluctuations in the yield of crops covering a large share of national cropland area have a greater impact on the stability of total national yield than fluctuations in the yield of crops occupying only small areas^22^.

In this study, we quantify the respective role of the average stability of individual crop yields and asynchrony among crop yield fluctuations on national yield stability. Following Renard and Tilman^16^, we compute total national yield stability as the ratio between the mean national yield (i.e. the summed annual kilocalories harvested across all edible crops in a nation, divided by the total area [ha] harvested) and the year-to-year standard deviation of this ratio. Although calories are not the most limiting nutrients in terms of nutrition at the global scale^23^, using caloric yields allow us to compare the production of the different crops grown within each nation. We further test whether fluctuations of the yield of the most abundant crops have a greater impact on the stability of total national yield. To do so, we calculate the average stability of yields of individual crops and asynchrony among crop yield fluctuations using two measures of crop yield, one non-weighted, and the other for which the yield is weighted by the proportion of total cropland it occupies (see Methods). Similarly, we quantify crop diversity by both crop richness and the exponential of the Shannon diversity index (H′) that is a measure of the effective diversity^24^. H′ weights each crop in a nation by the proportion of total cropland it occupies so that the crops that are most produced in a country count more than the minor crops in the calculation of diversity (see Methods). Finally, we assess by which of these mechanisms climate variability, crop diversity and agricultural inputs affect the stability of national food production.

## Results and discussion

We used structural equation models (SEMs) to disentangle the role of two stabilizing mechanisms (i.e. the average stability of individual crop yields and asynchrony) and three factors (i.e. climate variability, crop diversity and agricultural inputs) on national yield stability. We built two separate models, one based on crop richness, individual crop yield stability and asynchrony and the other based on these same indices weighted by the crop's share of total area harvested.

**Figure 1:**
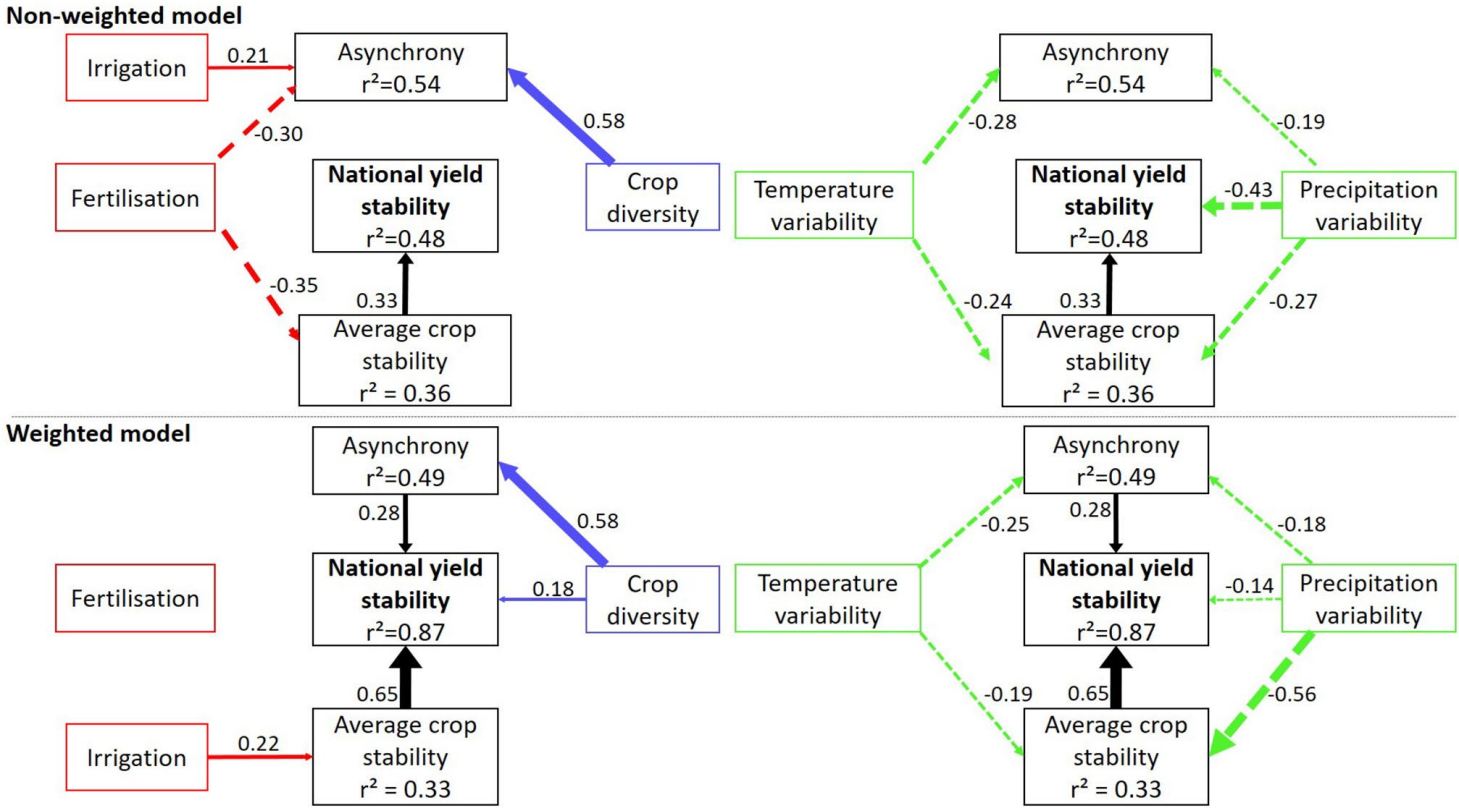
Structural equation model representing the drivers of the stability of national food production. For a seek of clarity, the contribution of agricultural inputs (red path) and crop diversity (blue path) to the stability of national food production are represented separately from the ones of climate variability (green path) [left and right panels, respectively]. Top: Non-weighted model. Crop diversity is quantified by crop richness. Down: Abundance weighted-model. Crop diversity is quantified by the exponential of Shannon diversity index. Asynchrony and average stability are weighted by the proportion of total cropland each crop occupied. The thickness of the arrows indicates the relative contribution of each variable. Plain arrows represent positive relationships while dotted arrows represent negative relationships between two boxes. Only relationships supported by the data (*P* > 0.05) are shown. Standardized regressions weights (along arrows) and squared coefficient of regressions (r^2^) for the fitting model are shown. Test indicates close model-data fit (Fisher’s C = 0.16, *P* = 0.92 and Fisher’s C = 0.46, *P* = 0.79 for non-weighted and weighed models, respectively). National yield stability, nitrogen use intensity and the percentage of land equipped for irrigation are log-transformed.

Our results reveal that the stability of national food production mainly depends on yield fluctuations of crops that cover a large proportion of the national harvested area. The model including the area-weighted measure of national crop yield explains 87% of the variance of national yield stability versus 48% for the model that does not account for differences in area planted for different crops (Fig. 1). This echoes recent findings showing that the fluctuations of the most largely produced crops have a larger weight on the stability of the total national food production^22^. Although crops with a lower share of cropland weight less in the process, their cultivation deliver other critical services for food security, for example by providing higher economic incomes or nutritional intakes^23,25^.

**Figure 2:**
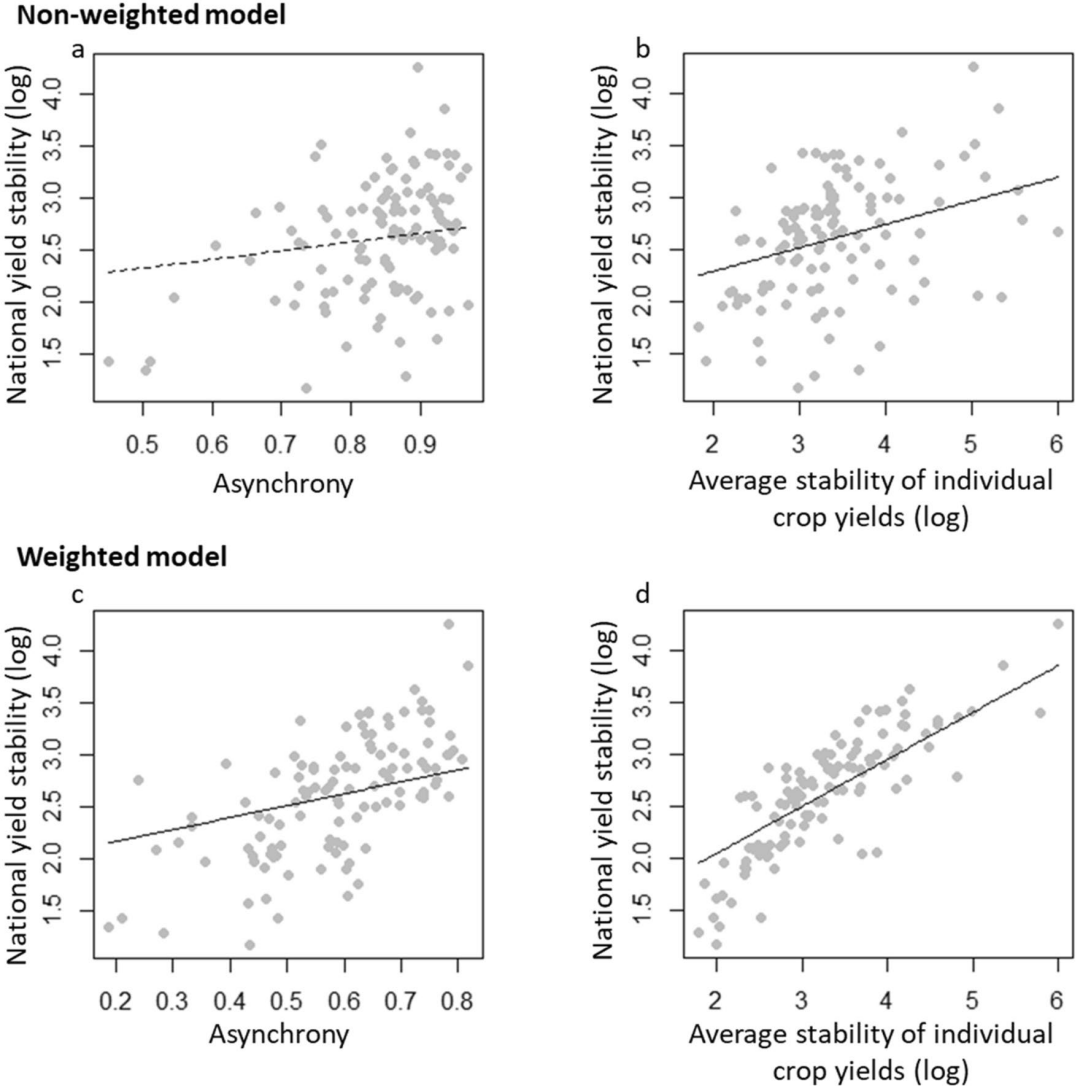
Effects of the average stability of individual crop yield and asynchrony on total, national yield stability. Black lines show the relationships estimated by the structural equation model when the crop yields are non-weighted (**a**, **b**) and weighted (**c**, **d**) by the proportion of cropland occupied by each crop within a country. Dashed lines represents non significant relationship.

Both the average stability of individual crop yield and yield asynchrony have significant, stabilizing effects on total national yield when yields are weighted by the proportion of area harvested (Figs. 1, 2). Importantly, this finding indicates that both fluctuations in the yield of a single crop and the dynamics that occur between crop yield fluctuations must be considered to ensure stable food production. However, the influence of the average stability of individual crop yields was twice as important as the one of asynchrony (Fig. 1). Conversely, when crop’s abundance is not accounted for, asynchrony has no significant effect on the stability of national yield (Fig. 1), suggesting that yield gains in crops representing low share of area harvested cannot compensate for yield losses in the most abundant crops at the national scale.

Climate variability has been identified as a main determinant of national food production instability^16^. Our results show that this destabilizing effect arises not only from a negative effect of precipitation and temperature variability on the average stability of individual crop yields, as previously observed^1^, but also from a synchronization of individual crop yield fluctuations (Fig. 1). This brings novel evidence that climate is a main driver of the synchronization of the global production of major commodities^4^.

**Figure 3:**
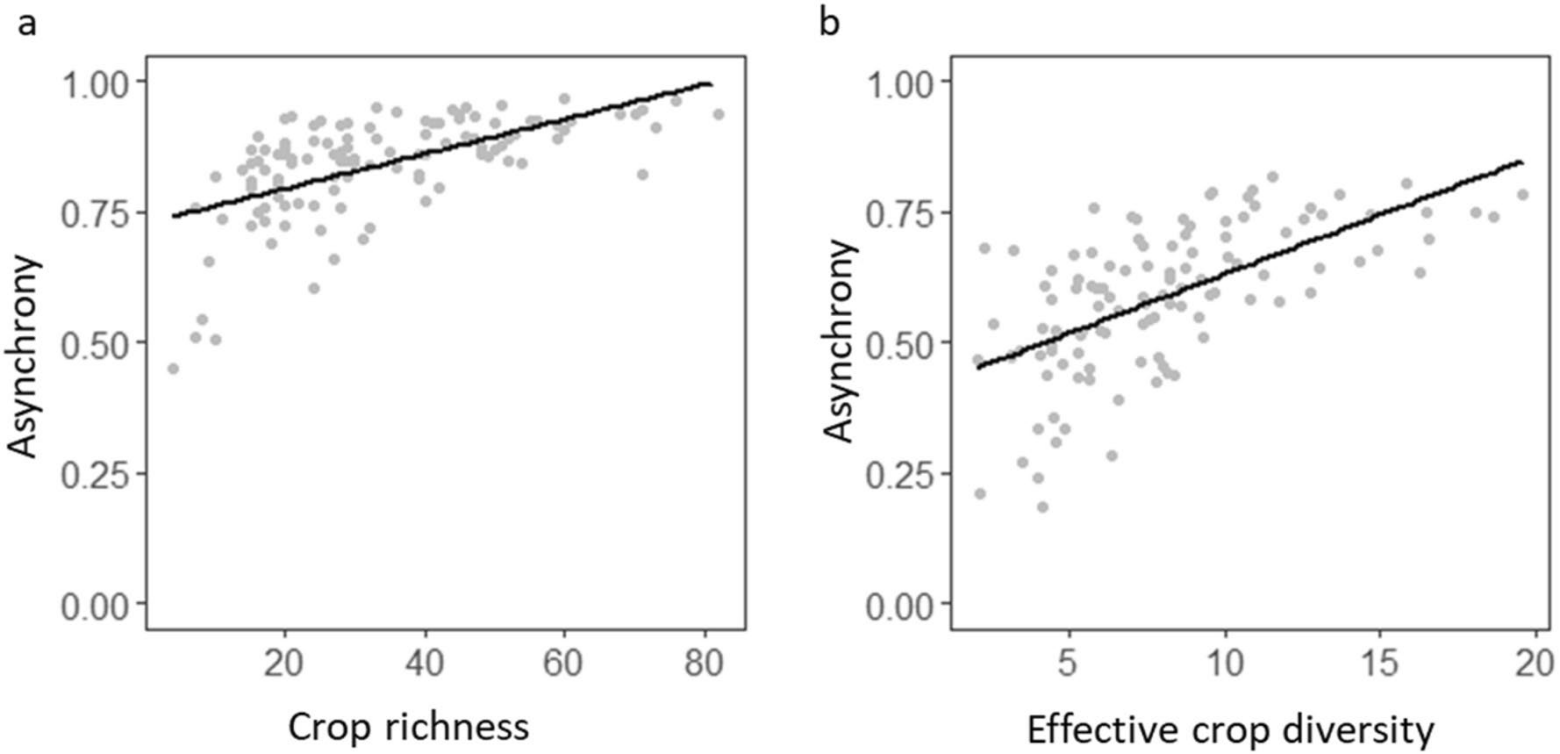
Effects of crop diversity on asynchronous dynamics among crop yield fluctuations. Black lines show the relationships estimated by the structural equation model when the crop yields are non-weighted (**a**) and weighted (**b**) by the proportion of cropland occupied by each crop within a country.

Previous studies have revealed the potential of crop diversity to increase the stability of crop production at the national scale^16,22^. Accordingly, we find a strong, positive effect of crop diversity on asynchrony (Fig. 3) that can largely counteract the synchronizing effect of climate variability (Fig. 1). This result indicates that asynchrony among the most abundant crops is the main process by which crop diversity stabilizes national food production^16^, confirming that the mechanisms invoked in BEF studies, classically conducted at local scales, can be extended to larger spatial scales^20,21^. However, whereas local-scale BEF studies often involve direct biotic interactions in driving asynchronous dynamics among species (e.g., ref.^19^), our dataset at national scale does not allow considerations of the effects of crop-crop interactions. Further research will be needed to go beyond our correlative approach to better understand the links between crop diversity, asynchrony and yield stability at such a large scale. In particularly, the role of spatial heterogeneity in environmental conditions in shaping the distribution of crops and in generating spatial asynchronous dynamics among different crops merits deeper exploration^20,21^.

**Figure 4:**
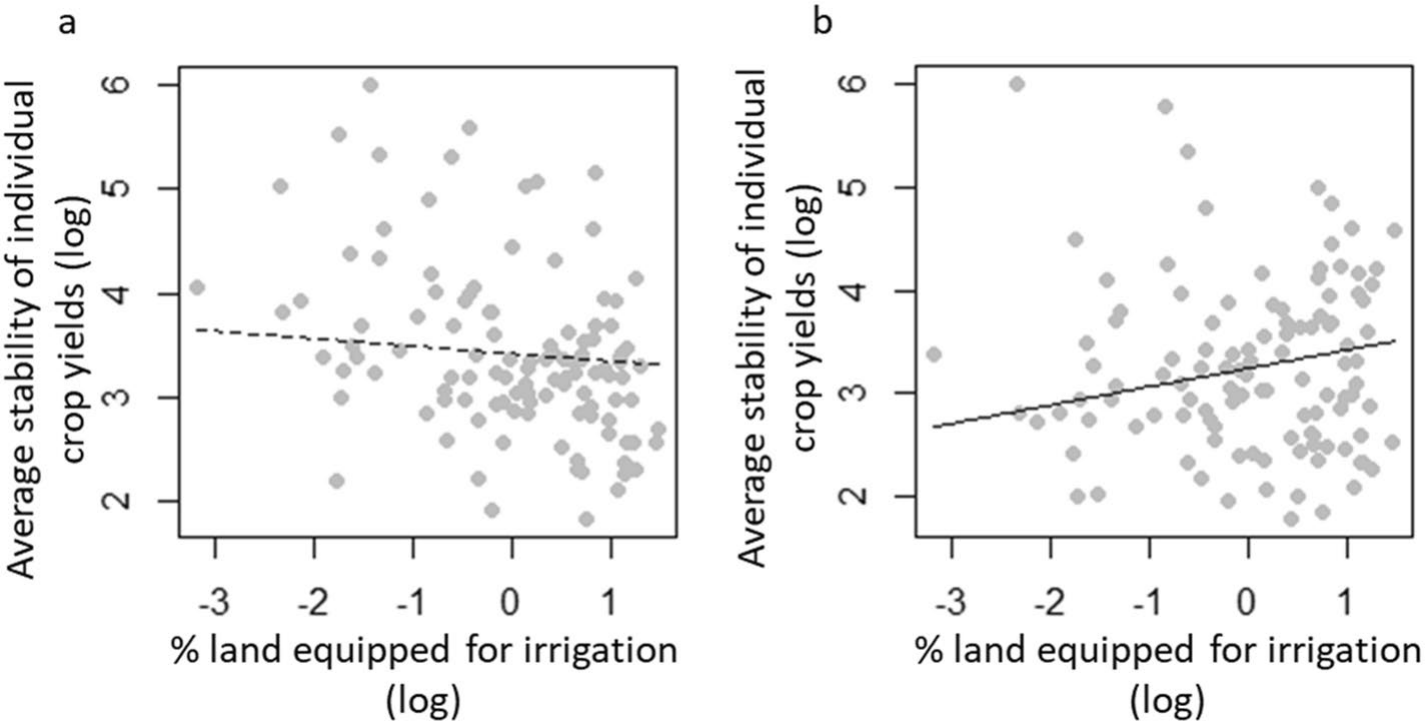
Effects of the percentage of land equipped for irrigation on the average stability of individual crop yields. Black lines show the relationships estimated by the structural equation model when the crop yields are non-weighted (**a**) and weighted (**b**) by the proportion of cropland occupied by each crop within a country.

Investigating the role of agricultural inputs shows that greater use of irrigation stabilizes total national food production by increasing the average stability of crops that occupy the largest proportion of the national harvested area (Figs. 1, 4). This probably reflects the fact that the most important crops for food consumption and trade (e.g. rice, cereals, maize) are also the most irrigated^26^. However, the stabilizing effect of irrigation on the yields of these crops is by far lower than the destabilizing one of climate variability (Fig. 1). The selection of varieties that are less reliant on irrigation and more resistant to climate variability will consequently be important to ensure a higher stability of crop yields^27–29^. Finally, we find that nitrogen fertilisation weakly affects the stability of the total, national yield although it is negatively associated with the average stability of individual crop yields when crop’s abundance is not accounted for (Fig. 1).

Ensuring the stability of food production at national level is becoming an increasingly important challenge for agricultural policies. Overall, our study reveals that both the average stability of individual crop yields and asynchrony in yield fluctuations among crops are important mechanisms to stabilize national food production in the face of climate variability. Promoting crop diversity at the national level might be a solution to promote multiple benefits, including greater stability of food production^16,22^, a diversified diet^30^ and the reduction of the use of agricultural inputs^31^. However, not all baskets of crops can provide all of these services, and our results suggest that it is important to design crop diversification strategies in a way that promotes asynchronous yield fluctuations between crops, for example by selecting species with different responses to environmental fluctuations. Finally, while the national level is the one at which agricultural policies are made, working at such a large scale does not allow to fully capturing determinants of food supply at finer scales. Transposing our approach to smaller scales will provide a better understanding of the determinants of stable food production worldwide.

## Materials and methods

### National yield stability

We used the FAOSTAT database (http://www.fao.org/faostat, visited in September 2019) to obtain data on annual crop production (in tons) and area harvested (in hectares) from 1961 to 2010 for 138 crops in 91 populous nations. Following Renard and Tilman^16^, we accounted for differences among nations in data quality and excluded five nations, namely North Korea, Guinea, Kenya, Mozambique and Zambia, for which at least 20% of the data on area harvested or production were extrapolated by the FAO (see details in^16^). We calculated for each nation and each year the total annual caloric yield (millions of kcal ha^-1^). To do so, we first calculated the kcal production of each crop by multiplying the production of each crop by its commodity-specific kilocalorie conversion factor from the USDA Nutrient Database^32^. In doing so, we were able to compare the production of different crops. Then, we summed these kcal harvests across all crops and divided this value by the sum of harvested area for all crops. We calculated national yield stability (*S*) as the ratio of mean total annual caloric yield (*µ*_*T*_) over its time-detrended standard deviation (*σ*_*T*_) for fifty consecutive years (1961–2010). We accounted for a temporal trend of increasing total annual crop yield by implementing a loess regression between annual crop yield and years. *σ*_*T*_ corresponds to the standard deviation of the residuals of this regression. Finally, we compared this stability index (largely used in the biodiversity-ecological functioning research, e.g.^14,16–18^) with the resilience index used by Zampieri et al.^22^. Both indices were strongly correlated (r = 0.992), strengthening our findings.

### Individual crop yield stability and yield asynchrony

For each country, we quantified the average stability of yields of individual crops as the mean of the inverse of the coefficient of variation of yield of each crop:

1 $$ {{\left( {\mathop \sum \limits_{i = 1}^{N} \frac{{\mu_{i} }}{{\sigma_{i} }}} \right)} \mathord{\left/ {\vphantom {{\left( {\mathop \sum \limits_{i = 1}^{N} \frac{{\mu_{i} }}{{\sigma_{i} }}} \right)} N}} \right. \kern-\nulldelimiterspace} N} $$

where $\mu_{i}$ is the temporal mean of crop’s annual kcal yield and $\sigma_{i}$ its time-detrended standard deviation. Time-detrended crop yield was computed through a loess regression between individual, annual crop yield and years.

We computed the asynchrony between crop yield fluctuations following the index developed by Loreau and De Mazancourt^11^:

2 $$ \Phi = 1 - \frac{{\sigma^{2}_{T} }}{{\left( {\mathop \sum \nolimits_{i = 1}^{N} \sigma_{i} } \right)^{2} }} $$

where Φ is the asynchrony of crop species based on annual caloric yield (millions of kcal ha^−1^) with $\sigma_{T}^{2}$ the temporal variance of the time-detrended national yield and $\sigma_{i}$ the time-detrended standard deviation of each crop’s annual kcal yield. The value of asynchrony varies between zero (perfect synchrony) and one (perfect asynchronous temporal fluctuations).

To test whether yield fluctuations of the most abundant crops have a greater impact on the stability of national food production, we weighted the annual yield of each crop by the proportion of total harvested area occupied by that crop. Average stability of yields of individual crops and yield asynchrony were computed on both the non-weighted and abundance-weighted yields.

### Crop diversity

For each country and year, we used both the total number of crop commodities (i.e. crop richness) and the Shannon information index (H′) to quantify crop diversity. H′ weights each crop in a nation by the proportion of total cropland it occupies (*p*_*i*_):

3 $$ H^{\prime } = - \mathop \sum \limits_{i = 1}^{N} \left( {p_{i} ln\left[ {p_{i} } \right]} \right) $$

with *N* being the total number of crops grown in a country each year.

The exponential form of the Shannon diversity index gives the effective crop diversity that is the number of crops representing an equal share of harvested area^24^. In other words, the exponential of the Shannon diversity index weighs all species by their frequency, without favouring either common or rare species^24^. We averaged the annual effective diversity of crop across the fifty years studied to test the effect of crop diversity on national yield stability.

### Agricultural inputs

We extracted the annual national application of nitrogen and the annual cropland area equipped for irrigation from the FAOSTAT database. Because Ireland, New Zealand and Netherlands use much of their fertilizers on pastures rather than croplands, we excluded these nations from our analysis. Similarly, we excluded Egypt because it has 100% of cropland equipped for irrigation. We calculated the annual rates of nitrogen application and irrigation per hectare by dividing their use by the total annual cropland area.

### Climate variability

We used global gridded climatic data from the Climate Research Unit of the University of East Anglia^33^ to compute the year-to-year variability of growing season precipitation and temperature for each country, both strongly affecting the stability of national food production^16^. From these data, we derived annual precipitation and temperature for each grid cell in a country by taking the sum of monthly precipitation and the mean of monthly temperature values weighted by the proportion of cropland in each grid cell^34^. We then computed the year-to-year coefficient of variation of cropland-based temperature and precipitation for each country.

### Statistical analysis

We used structural equation models (SEMs) to evaluate how irrigation, intensity of use of nitrogen fertilizers and crop diversity affected national yield stability through changes in the average stability of yields of individual crops and asynchrony of yields. SEMs represent a powerful way to disentangle complex mechanisms controlling crop diversity-stability relationships, as previously done in natural ecosystems (e.g.^14,15,35,36^). We set up two different structural equation models, one based on non-weighted indices of stability of individual crops and asynchrony, the other based on the same indices weighted by the proportion of total harvested area accounted for by each crop. We firstly considered the effects of agricultural inputs and crop diversity on the stability of national food production via the path of average yield stability. The second path quantified the indirect effects of agricultural inputs and crop diversity on national stability via their impacts on crop yield asynchrony. We also accounted for the direct effects of agricultural inputs and crop diversity on national yield stability. Finally, we controlled for the effects of climate variability on total, national yield stability, individual crop yield stability and yield asynchrony. SEMs were run with the lavaan R library^37^. We used the standardized estimates to compare the relative importance of the different paths. The model fit was evaluated using the Fisher C’score and its associated *p* values. Because the structural equation model assumes linear relationships between predictors and the dependent variable, we also plotted the relationships between total national yield stability and both asynchrony and average stability of individual crop yield to control for linearity (Fig. 2). Similarly, we investigated the relationships between crop diversity and asynchrony (Fig. 3), as well as between irrigation rate and the average stability of individual crop yield (Fig. 4).

## Data Availability

The sources of all data used in this study are referenced in the "Materials and methods" section and all raw data are freely accessible at the URLs provided. The datasets used for the analyses are available from the corresponding author upon request.

## References

[1] Brown JKM (2019). Yield instability of winter oilseed rape modulated by early winter temperature. Sci. Rep..

[2] Lesk C (2016). Influence of extreme weather disasters on global crop production. Nature.

[3] Ray DK (2015). Climate variation explains a third of global crop yield variability. Nat. Commun..

[4] Mehrabi Z, Ramankutty N (2019). Synchronized failure of global crop production. Nat. Ecol. Evol..

[5] Schmidhuber J, Tubiello FN (2007). Global food security under climate change. Proc. Natl. Acad. Sci. U. S. A..

[6] Laio F (2016). The past and future of food stocks. Environ. Res. Lett..

[7] D’Odorico P (2014). Feeding humanity through global food trade. Earths Future.

[8] Marchand P (2016). Reserves and trade jointly determine exposure to food supply shocks. Environ. Res. Lett..

[9] Puma MJ (2015). Assessing the evolving fragility of the global food system. Environ. Res. Lett..

[10] d’Amour CB, Anderson W (2020). International trade and the stability of food supplies in the Global South. Environ. Res. Lett..

[11] Loreau M, de Mazancourt C (2008). Species synchrony and its drivers: neutral and nonneutral community dynamics in fluctuating environments. Am. Nat..

[12] Herrera JM (2020). Lessons from 20 years of studies of wheat genotypes in multiple environments and under contrasting production systems. Front. Plant Sci..

[13] Liu J (2019). Nitrogen addition reduced ecosystem stability regardless of its impacts on plant diversity. J. Ecol..

[14] Zhang Y (2016). Nitrogen enrichment weakens ecosystem stability through decreased species asynchrony and population stability in a temperate grassland. Glob. Change Biol..

[15] Song M-H (2019). Nutrient-induced shifts of dominant species reduce ecosystem stability via increases in species synchrony and population variability. Sci. Total Environ..

[16] Renard D, Tilman D (2019). National food production stabilized by crop diversity. Nature.

[17] Loreau M, de Mazancourt C (2013). Biodiversity and ecosystem stability: a synthesis of underlying mechanisms. Ecol. Lett..

[18] Thibaut LM, Connolly SR (2013). Understanding diversity-stability relationships: towards a unified model of portfolio effects. Ecol. Lett..

[19] Tilman D (2006). Biodiversity and ecosystem stability in a decade-long grassland experiment. Nature.

[20] Gonzalez A (2020). Scaling-up biodiversity-ecosystem functioning research. Ecol. Lett..

[21] Wang S, Loreau M (2016). Biodiversity and ecosystem stability across scales in metacommunities. Ecol. Lett..

[22] Zampieri M (2020). Estimating resilience of crop production systems: From theory to practice. Sci. Total Environ..

[23] Kc KB (2018). When too much isn’t enough: Does current food production meet global nutritional needs?. PLoS One.

[24] Jost L (2006). Entropy and diversity. Oikos.

[25] Cheng A (2017). Diversifying crops for food and nutrition security—a case of teff. Biol. Rev..

[26] FAO Aquastat. http://www.fao.org/aquastat/en/geospatial-information/global-maps-irrigated-areas.

[27] Harfouche AL (2019). Accelerating climate resilient plant breeding by applying next-generation artificial intelligence. Trends Biotechnol..

[28] Kahiluoto H (2019). Decline in climate resilience of European wheat. Proc. Natl. Acad. Sci. U. S. A..

[29] Zhang H (2018). Developing naturally stress-resistant crops for a sustainable agriculture. Nat. Plants.

[30] Herrero M (2017). Farming and the geography of nutrient production for human use: a transdisciplinary analysis. Lancet Planet. Health.

[31] Isbell F (2017). Benefits of increasing plant diversity in sustainable agroecosystems. J. Ecol..

[32] FoodData Central. [Online]. Available: https://fdc.nal.usda.gov/. Accessed: 27-May-2020.

[33] Harris I (2020). Version 4 of the CRU TS monthly high-resolution gridded multivariate climate dataset. Sci. Data.

[34] Ramankutty N, Evan AT, Monfreda C, Foley JA (2008). Farming the planet: 1. Geographic distribution of global agricultural lands in the year 2000. Glob. Biogeochem. Cycle.

[35] Bluethgen N (2016). Land use imperils plant and animal community stability through changes in asynchrony rather than diversity. Nat. Commun..

[36] Shi Z (2016). Dual mechanisms regulate ecosystem stability under decade-long warming and hay harvest. Nat. Commun..

[37] Rosseel, Y. *et al.**lavaan: Latent Variable Analysis* (2020).

